# Integrating Bioethics and Science Communication Insights to Promote Translational Justice, Critical Consciousness, and Global Health Equity in COVID-19 Vaccination

**DOI:** 10.1080/15265161.2025.2497987

**Published:** 2025-06-06

**Authors:** John Noel Montaño Viaña, Merryn McKinnon, Carys Fisser

**Affiliations:** The Australian National University

The publications of Allyse et al. (2025) and Geneviève et al. (2025) advocate for justice and equity in healthcare and explore how bioethical insights can be enriched by critical discourse from other disciplines. Allyse et al. (2025) draw from science and technology studies and sociology to develop a translational justice tool for health equity. They underscore the importance of incorporating social values, acknowledging end-user perspectives, addressing access barriers, and reforming success metrics in biomedical innovation to minimize inequitable outcomes from resulting technologies. Geneviève et al. (2025) integrate core tenets from critical race theory and critical gerontology to create an analytical framework for public health interventions. Their ARIE (Age- and Race-conscious Interventions done Equitably) framework encourages critical consciousness in assessing the impacts of public health measures on older racialized minorities, recognizing multiple systemic and institutionalized forms of discrimination experienced by intersectionally minoritised and/or marginalized groups. Both Allyse et al. (2025) and Geneviève et al. (2025) used examples during the COVID-19 pandemic to illustrate how testing, lockdowns, epidemiologic reporting, and vaccine development have failed to account for certain populations, leading to poor health outcomes and distrust in public health systems.

We commend the engagement of Allyse et al. (2025) and Geneviève et al. (2025) with various disciplines to bring critical insights to bioethics, widening the field’s gaze to account for historical injustices, structural inequities, and continuing marginalization experienced by various populations. Translational justice and critical consciousness have definite crucial roles to play in encouraging more equitable biomedical innovation and public health promotion. Given that both papers focused on European and North American contexts, it is worth reflecting on how these insights can be applied to examine and address regional, or even global, disparities. Furthermore, what additional disciplinary tools could further enrich the conceptualization and application of critical bioethics?

This commentary reflects on the potential contribution of science communication perspectives in mobilizing translational justice and critical consciousness movements to achieve global health equity. While the definition of science communication varies depending on particular goals and contexts (Kappel and Holmen 2019), here we define it as the practice of sharing science-based information with the intent of achieving a specific goal, such as promoting its uptake or collaboratively examining its relevance and associated ethical and societal ramifications. We propose that science communication could meaningfully work in tandem with bioethics to examine and address issues in health-related contexts. To illustrate the value of this cross-disciplinary collaboration, we first present key findings from a preliminary review of COVID-19 vaccine equity discourses in leading bioethics and science communication journals, highlighting how insights from both disciplines can be synthesized to account for more diverse populations, contexts, and socio-econo-cultural conditions. We then discuss how science communication insights and methods can help bioethics enrich, extend, and enact its normative recommendations, making it walk its talk.

## BIOETHICS AND SCIENCE COMMUNICATION INSIGHTS ON COVID-19 VACCINE EQUITY

We systematically mapped (James, Randall, and Haddaway 2016) publications on COVID-19 vaccination equity in five leading bioethics (*Nursing Ethics, BMC Medical Ethics, Journal of Medical Ethics, The American Journal of Bioethics, and Hastings Center Report*) and five science communication-related journals (*Public Understanding of Science*, *Science Communication*, *Journal of Science Communication, Frontiers in Communication,* and *Humanities and Social Sciences Communication - HSSC*). The top bioethics journals were determined using their averaged discipline-specific rank in Scimago, Clarivate (Medical Ethics), and Google Scholar. Science communication has a much smaller number of journals, with only the first three included being discipline specific. While *Frontiers* and *HSSC* publish on a broader range of topics, they do have specific relevance to science communication and are included to capture a broad sample of science communication research. The search date was restricted to 1 March 2020 to 31 July 2022 to cover the period where COVID-19 was declared a pandemic and the production and roll out of various COVID-19 vaccines. The search terms “COVID”, “COVID-19”, “coronavirus”, “equity”, and “vaccination” were used to retrieve articles from the journal websites. We then reviewed the title, abstract, keywords, and full texts of retrieved articles to confirm that that they focus on COVID, equity, and vaccination. Several equity-related keywords were also collaboratively generated to aid with screening for relevant articles: Equit*, Just*, Access*, Disparit*, Minorit*, Marginalis*, Vulnerab*, Intersection*, Divers*, Inequalit*, Inclusi*, and Exclusi*. Findings of/arguments in relevant articles were independently coded to examine the focus of the research in terms of (1) scale (local, national, or global); (2) population; and (3) type of equity. All authors discussed and agreed upon the final coding of all articles.

**Figure 1.**
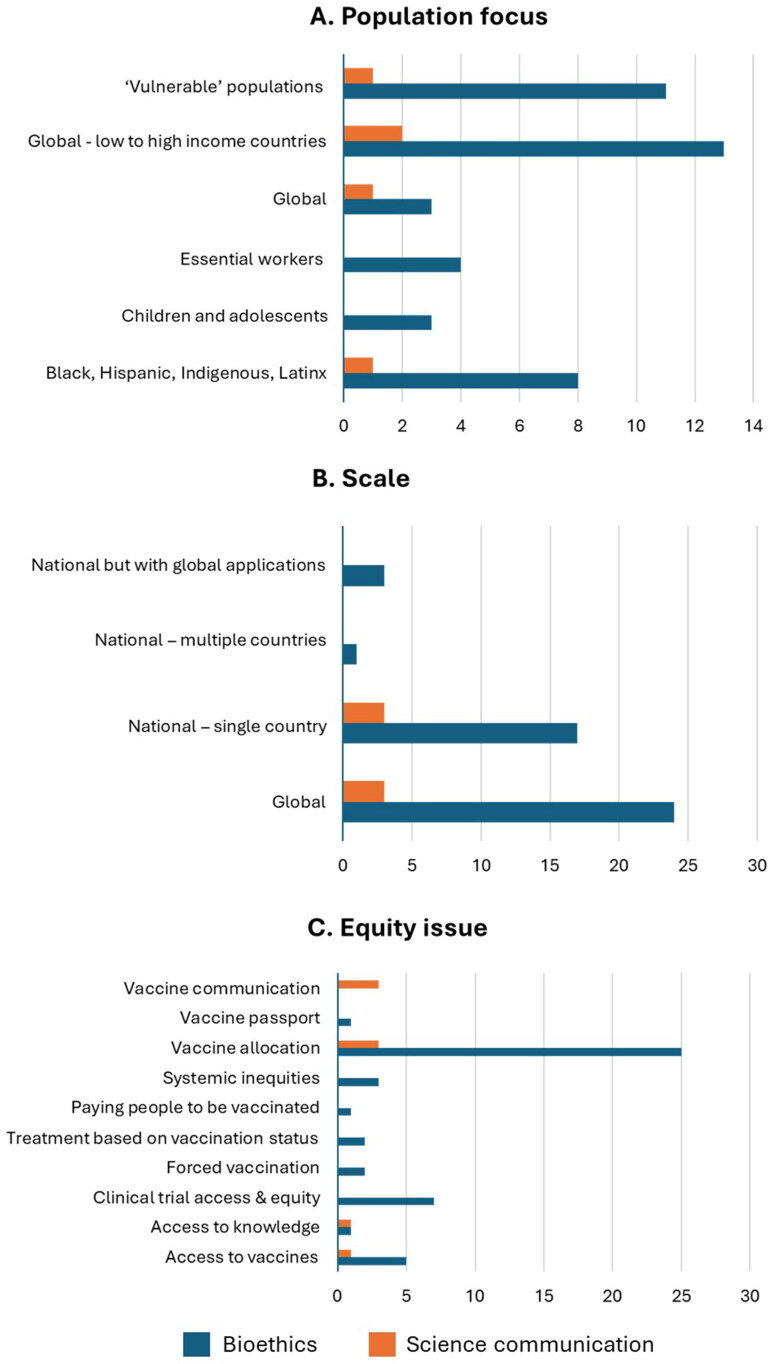
(A) Population/audience, (B) Scale, and (C) Equity issue of focus of bioethics and science communication articles included in the final sample (*n* = 51). Some articles focus on more than one equity issue.

The initial search retrieved 556 articles, 51 of which were deemed relevant and were coded. Of the 51 articles, 45 are in bioethics journals and six articles are in three science communication journals (*Science Communication, Humanities and Social Sciences Communication,* and *Frontiers in Communication*). While Allyse et al. (2025) and Geneviève et al. (2025) discussed disparities and/or exclusion experienced by older racial minorities and pregnant women and primarily focused on the Global North, our systematic map revealed a wider range of populations (Figure 1(A)) and contexts (Figure 1(B)) that can be impacted by inequities in vaccine research, distribution, and communication. Particular populations have been identified in terms of potential or experienced inequities, with several papers broadly categorizing them as “vulnerable populations” and with others specifying concerns surrounding children/adolescents, racial/ethnic minorities, and/or essential workers (Figure 1(A)). Our systematic mapping also highlights other populations that could experience disparities, including First Nation communities (Torrie et al. 2021); incarcerated individuals (James 2022); people with developmental and intellectual disabilities (Shevzov-Zebrun and Caplan 2022); and other intersectionally marginalized groups such as children from low socio-economic backgrounds and ethnic minorities in low and middle-income countries (Brusa and Barilan 2021).

Papers also examined issues at varying scales (Figure 1(B)), with bioethics journals publishing more papers on the global, rather than the national, scale. Examination of global inequities highlighted disparities between high and low to middle-income countries. Thus, our systematic mapping underscores how translational justice and critical consciousness should also account for the global political economy, such as Central Asian countries acquiring vaccines from Russia and China (Aripov et al. 2022) and booster provision in wealthy countries impacting primary vaccination in less affluent nations (Jecker and Lederman 2021). Science communication articles advocate for global vaccine equity (Rydland et al. 2022), underscoring the need for coordinated interdisciplinary and international efforts (Vuong et al. 2022) and the role of social media in advocating for vaccine diplomacy to increase supplies in low to middle-income countries (Yin et al. 2022).

In terms of equity issues discussed, bioethics publications primarily underscored issues relating to vaccine allocation, access, and clinical trial participation. Science communication publications also explored vaccine allocation issues, followed by concerns regarding communication and access to knowledge (Figure 1(C)). These findings, particularly science communication publications that discuss epistemic and informational disparities, illustrate potential gaps in bioethical discourses. Science communication papers typically dealt with dis- and misinformation (Vuong et al. 2022), risk perceptions (Boyd and Buchwald 2022), and vaccine hesitancy (Yin et al. 2022). They also discussed how certain groups were excluded from any form of communication about COVID-19 vaccines, simply because of their minoritised status within a community (van den Muijsenbergh et al. 2022). These highlight the need for public health programs to ensure that interventions such as vaccines do not just reach diverse populations, but that these populations also receive sufficient information on the interventions being promoted, along with their risks and benefits.

## SCIENCE COMMUNICATION FOR ENRICHING, ENACTING, AND EXTENDING BIOETHICAL RECOMMENDATIONS

Findings from our systematic mapping demonstrate how science communication could enrich bioethical insights, particularly on the impact of epistemic and informational disparities on health equity. It is inherently understood within science communication that simply having access to a resource is not necessarily the key determining factor in whether someone will choose to use or accept it, particularly vaccines (Thaker, Richardson, and Holmes 2023). If people are exposed to misinformation and disinformation regarding COVID-19 vaccines (Cagayan, Mendoza, and Viana 2022), do not have access to the proper information channels, or do not receive balanced and up-to-date information to make well-informed decisions, then they may still hesitate to get vaccinated. Bioethics paradigms on translational justice and critical consciousness therefore need to account for epistemic systems surrounding health promotion programs or biomedical technologies, as they could help identify populations who might not be willing to receive, and thus might be excluded in benefitting from, particular interventions.

In addition to enriching bioethics discussions, we also believe in the role of science communication in enacting normative recommendations and in extending their reach beyond philosophers, scientists, and health professionals. Bioethicists could draw from science communication methods and modalities for engaging with diverse publics and stakeholders (Orthia et al. 2021). Science communication researchers have also long advocated against the deficit model in public engagement, where the public are seen as passive recipients of information. Similarly, this could also inspire bioethicists to be less paternalistic (Viaña, Raman, and Barber 2021) in making normative recommendations. Instead, they could closely and humbly engage with marginalized communities (Orthia et al. 2021; Kappel and Holmen 2019) and co-develop recommendations with them, as opposed to just thinking or theorizing what is or would be best for them (Viaña, Raman, and Barber 2021). Accounting for diverse values, perspectives, positionalities, and contexts could enable the formulation of more pragmatic normative recommendations that minoritised groups, decision makers, healthcare providers, and biomedical researchers can readily adopt and apply. Ultimately, closer collaboration between bioethicists and science communicators could enable critical insights from both disciplines to permeate public and political discourse, mobilizing multiple sectors to actively tackle local, national, and global health inequities.

Overall, science communication and bioethics are well positioned to provide complementary perspectives on social processes influencing disease burdens, interventions, and outcomes globally. Bioethics can explore many of the ethical issues inherent in the structural systems and processes that influence epistemic production and technological distribution; whereas science communication is well placed to explore societal perceptions and uptake of scientific information and products. Promising initiatives in bioethics communication, such as the Dracopoulos-Bloomberg iDeas Lab at the Johns Hopkins Berman Institute of Bioethics (Rieder, Lauren Arora, and Kahn 2022), can provide a model for this multi-disciplinary collaboration and its application for greater engagement with scientists, policy makers, community leaders, and diverse publics. We believe it is time to move beyond disciplinary silos. Bioethics, science communication, and other social science disciplines could collaborate more in promoting inclusive, equitable, and just responses to structural health inequities, particularly those experienced by minoritised communities in multicultural societies and by the global majority in Global South countries.
